# Supplementary material for: Rapid coral reef assessment using 3D modelling and acoustics: acoustic indices correlate to fish abundance, diversity and environmental indicators in West Papua, Indonesia
Source: PeerJ. 2021 Feb 8;9:e10761. doi: 10.7717/peerj.10761 (PMC7877240; doi:10.7717/peerj.10761)
Supplement: Supplemental Information 1 [file peerj-09-10761-s001.pdf]

S1. Correlation analysis for temporal stability of Acoustic indices over time

Outputs for correlation coefficients and significance for each acoustic index when comparing;

1. Minutes 4 – 5
2. Minutes 5 – 6
3. Minutes 6 – 7
4. Minutes 4 – 7
5. The average value of 1 to 3 above

Note: Significance values of 0 refer to $p<0.00001$. Figures indicate correlation (Direction as colour) and size as correlation coefficient. Blank squares show no significant correlation.

AD High

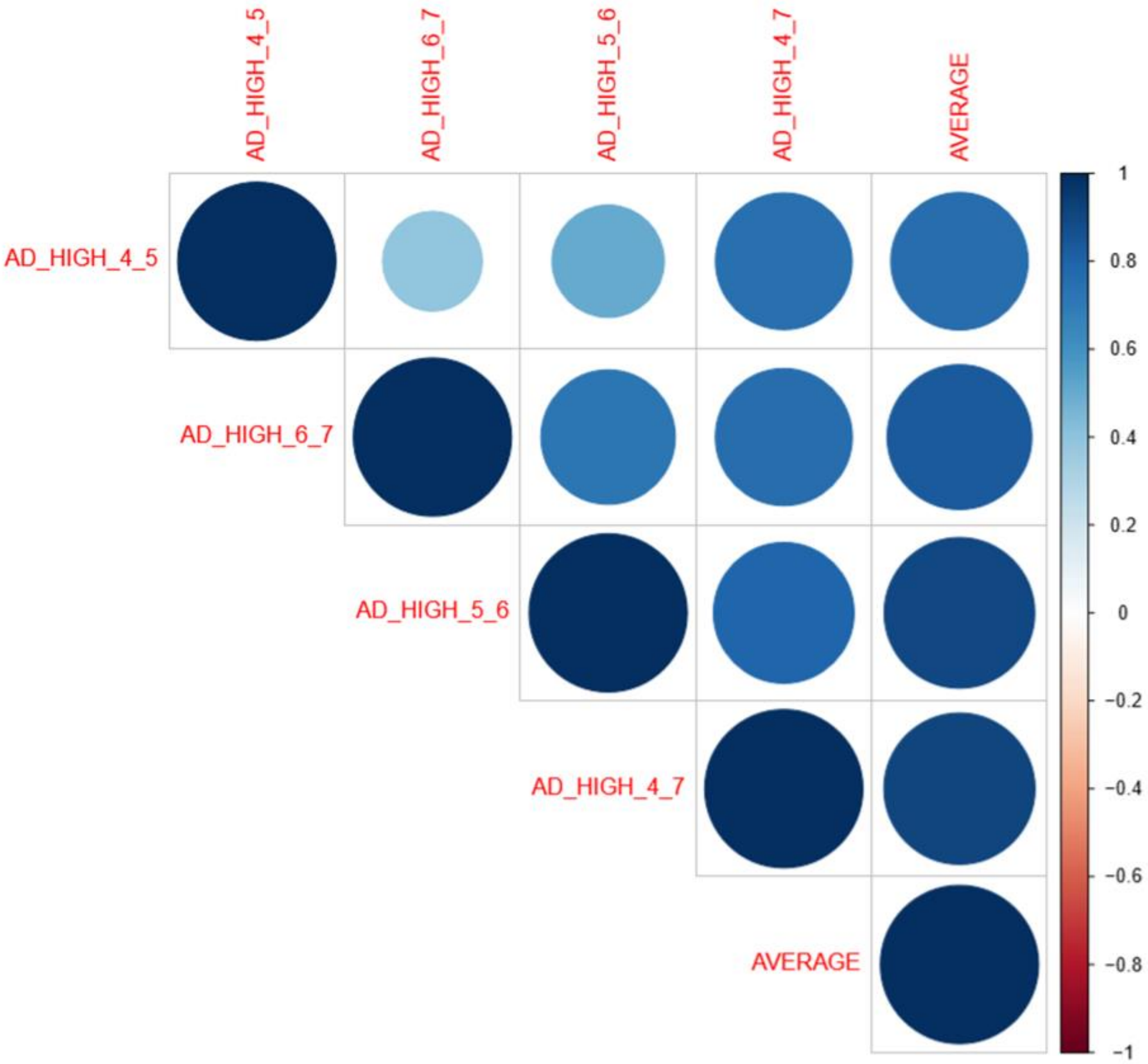

Correlation Coefficients

	AD_HIGH_4_5	AD_HIGH_5_6	AD_HIGH_6_7	AD_HIGH_4_7	AVERAGE
AD_HIGH_4_5	1.00	0.50	0.40	0.75	0.75
AD_HIGH_5_6	0.50	1.00	0.72	0.79	0.91
AD_HIGH_6_7	0.40	0.72	1.00	0.75	0.84
AD_HIGH_4_7	0.75	0.79	0.75	1.00	0.91
AVERAGE	0.75	0.91	0.84	0.91	1.00

p-values

	AD_HIGH_4_5	AD_HIGH_5_6	AD_HIGH_6_7	AD_HIGH_4_7	AVERAGE
AD_HIGH_4_5		0.0024	0.0193	0.0000	0.0000
AD_HIGH_5_6	0.0024		0.0000	0.0000	0.0000
AD_HIGH_6_7	0.0193	0.0000		0.0000	0.0000
AD_HIGH_4_7	0.0000	0.0000	0.0000		0.0000
AVERAGE	0.0000	0.0000	0.0000	0.0000	

AD Low

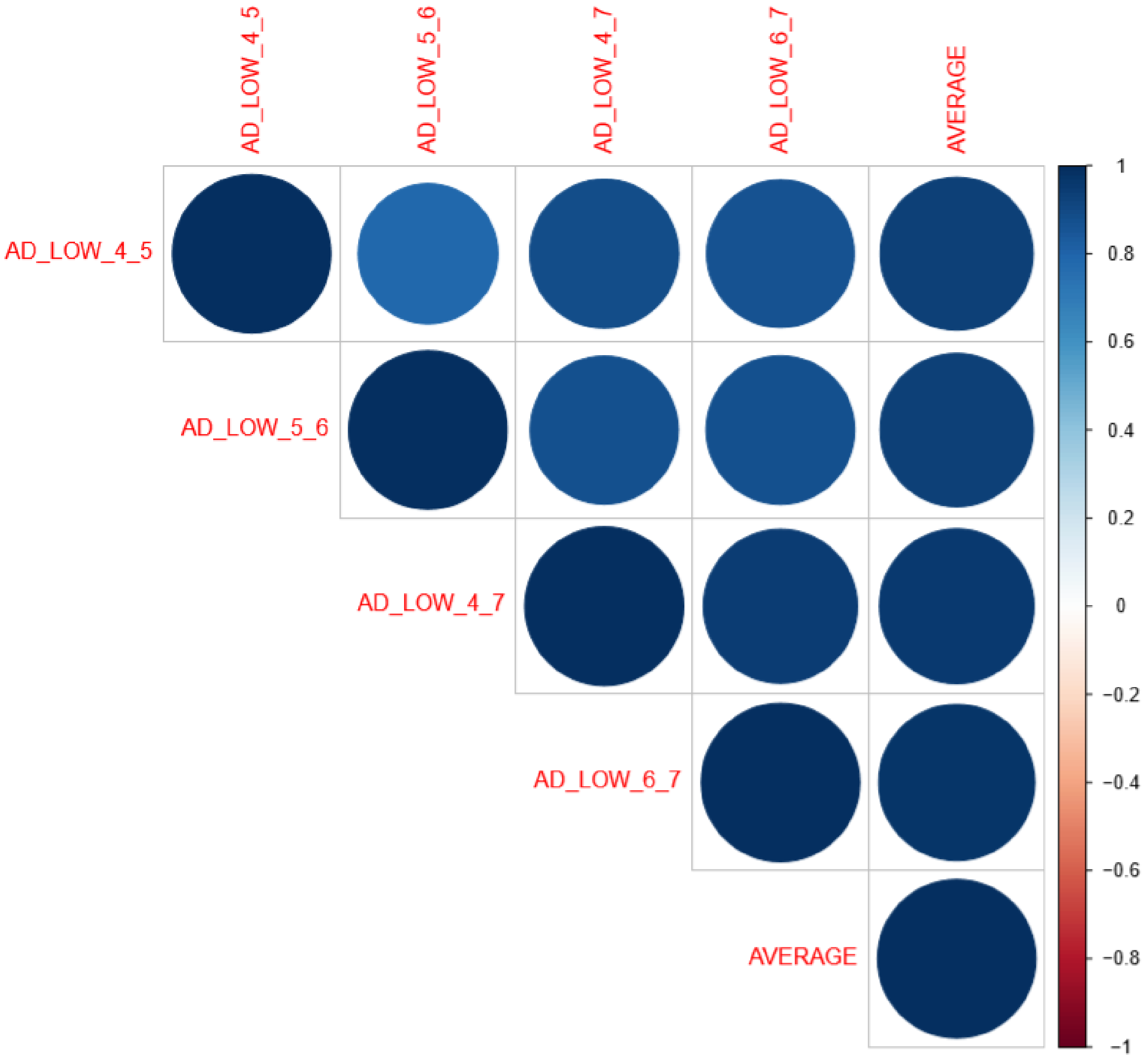

Correlation Coefficients

	AD_LOW_4_5	AD_LOW_5_6	AD_LOW_6_7	AD_LOW_4_7	AVERAGE
AD_LOW_4_5	1.00	0.78	0.87	0.88	0.93
AD_LOW_5_6	0.78	1.00	0.88	0.87	0.93
AD_LOW_6_7	0.87	0.88	1.00	0.94	0.97
AD_LOW_4_7	0.88	0.87	0.94	1.00	0.95
AVERAGE	0.93	0.93	0.97	0.95	1.00

p-values

	AD_LOW_4_5	AD_LOW_5_6	AD_LOW_6_7	AD_LOW_4_7	AVERAGE
AD_LOW_4_5		0	0	0	0
AD_LOW_5_6	0		0	0	0
AD_LOW_6_7	0	0		0	0
AD_LOW_4_7	0	0	0		0
AVERAGE	0	0	0	0	

AD Ratio

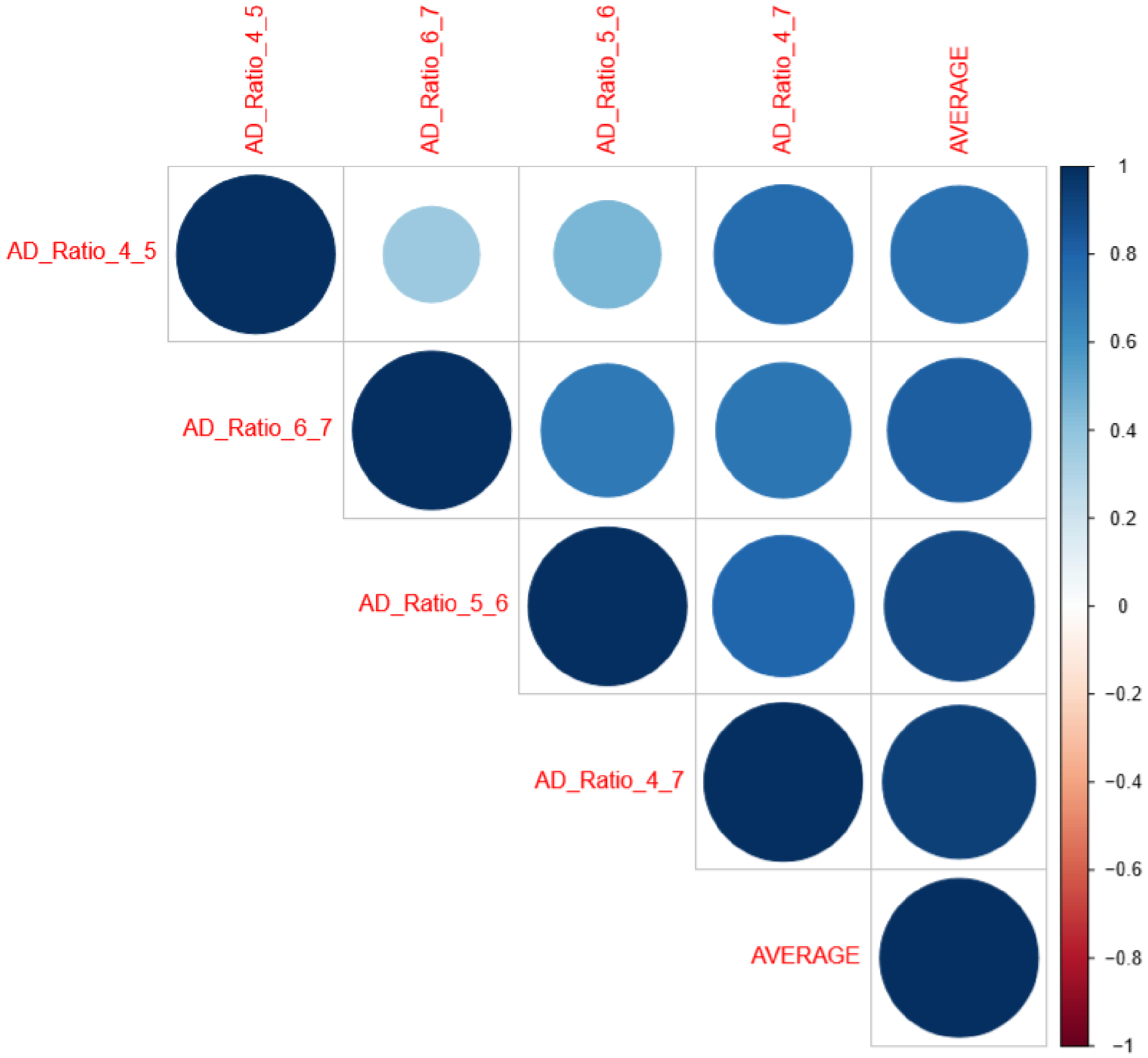

	AD_Ratio_4_5	AD_Ratio_5_6	AD_Ratio_6_7	AD_Ratio_4_7	AVERAGE
AD_Ratio_4_5	1.00	0.46	0.37	0.77	0.75
AD_Ratio_5_6	0.46	1.00	0.70	0.79	0.89
AD_Ratio_6_7	0.37	0.70	1.00	0.73	0.82
AD_Ratio_4_7	0.77	0.79	0.73	1.00	0.93
AVERAGE	0.75	0.89	0.82	0.93	1.00

p-values

	AD_Ratio_4_5	AD_Ratio_5_6	AD_Ratio_6_7	AD_Ratio_4_7	AVERAGE
AD_Ratio_4_5		0.0066	0.0316	0.0000	0.0000
AD_Ratio_5_6	0.0066		0.0000	0.0000	0.0000
AD_Ratio_6_7	0.0316	0.0000		0.0000	0.0000
AD_Ratio_4_7	0.0000	0.0000	0.0000		0.0000
AVERAGE	0.0000	0.0000	0.0000	0.0000	

AEI High

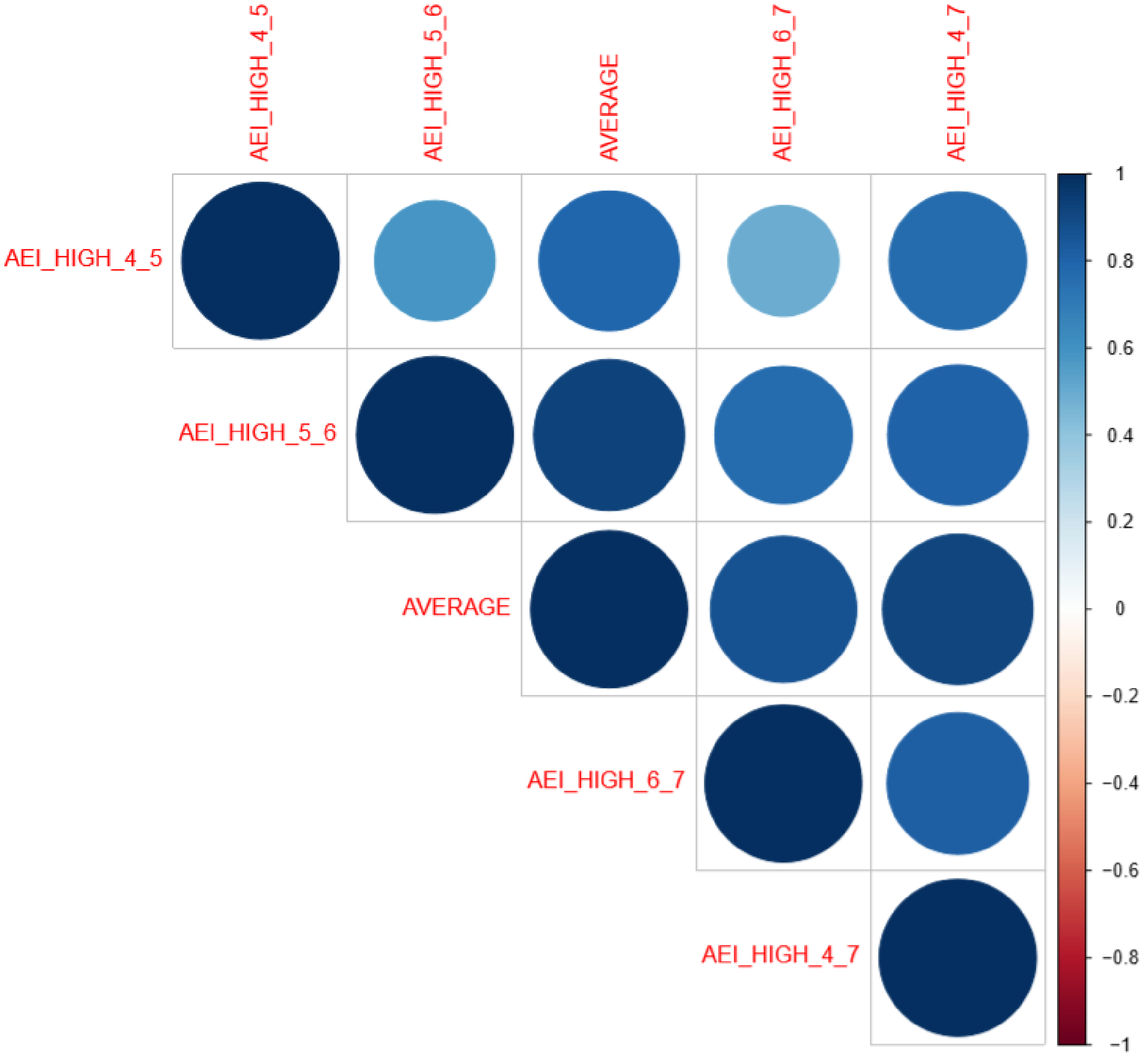

Correlation Coefficients

	AEI_HIGH_4_5	AEI_HIGH_5_6	AEI_HIGH_6_7	AEI_HIGH_4_7	AVERAGE
AEI_HIGH_4_5	1.00	0.59	0.50	0.77	0.79
AEI_HIGH_5_6	0.59	1.00	0.77	0.80	0.93
AEI_HIGH_6_7	0.50	0.77	1.00	0.81	0.87
AEI_HIGH_4_7	0.77	0.80	0.81	1.00	0.92
AVERAGE	0.79	0.93	0.87	0.92	1.00

p-values

	AEI_HIGH_4_5	AEI_HIGH_5_6	AEI_HIGH_6_7	AEI_HIGH_4_7	AVERAGE
AEI_HIGH_4_5		0.0003	0.0026	0.0000	0.0000
AEI_HIGH_5_6	0.0003		0.0000	0.0000	0.0000
AEI_HIGH_6_7	0.0026	0.0000		0.0000	0.0000
AEI_HIGH_4_7	0.0000	0.0000	0.0000		0.0000
AVERAGE	0.0000	0.0000	0.0000	0.0000	

AEI Low

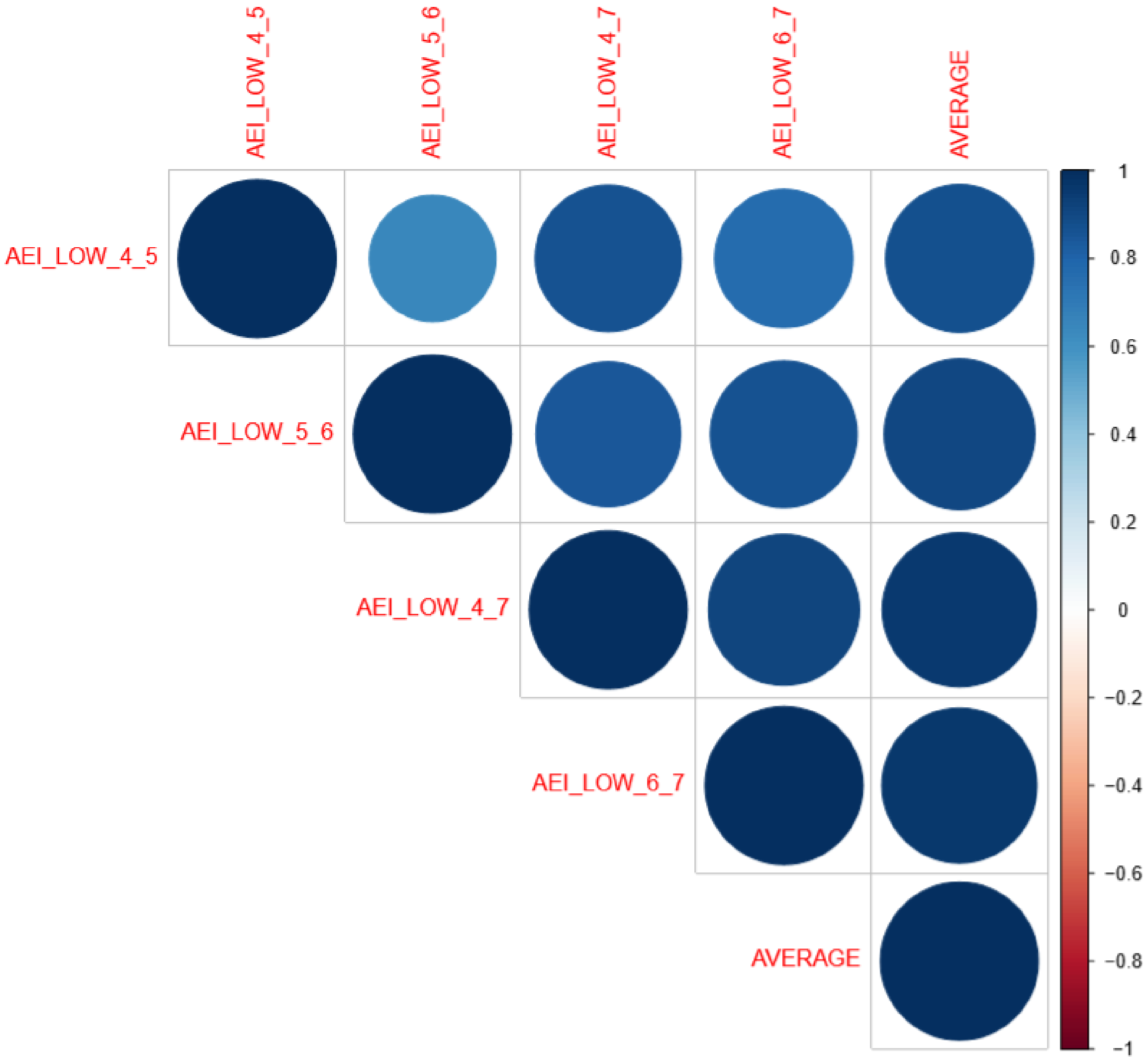

Correlation Coefficients

	AEI_LOW_4_5	AEI_LOW_5_6	AEI_LOW_6_7	AEI_LOW_4_7	AVERAGE
AEI_LOW_4_5	1.00	0.64	0.77	0.86	0.87
AEI_LOW_5_6	0.64	1.00	0.86	0.84	0.91
AEI_LOW_6_7	0.77	0.86	1.00	0.92	0.96
AEI_LOW_4_7	0.86	0.84	0.92	1.00	0.95
AVERAGE	0.87	0.91	0.96	0.95	1.00

p-values

	AEI_LOW_4_5	AEI_LOW_5_6	AEI_LOW_6_7	AEI_LOW_4_7	AVERAGE
AEI_LOW_4_5		0	0	0	0
AEI_LOW_5_6	0		0	0	0
AEI_LOW_6_7	0	0		0	0
AEI_LOW_4_7	0	0	0		0
AVERAGE	0	0	0	0	

AEI Ratio

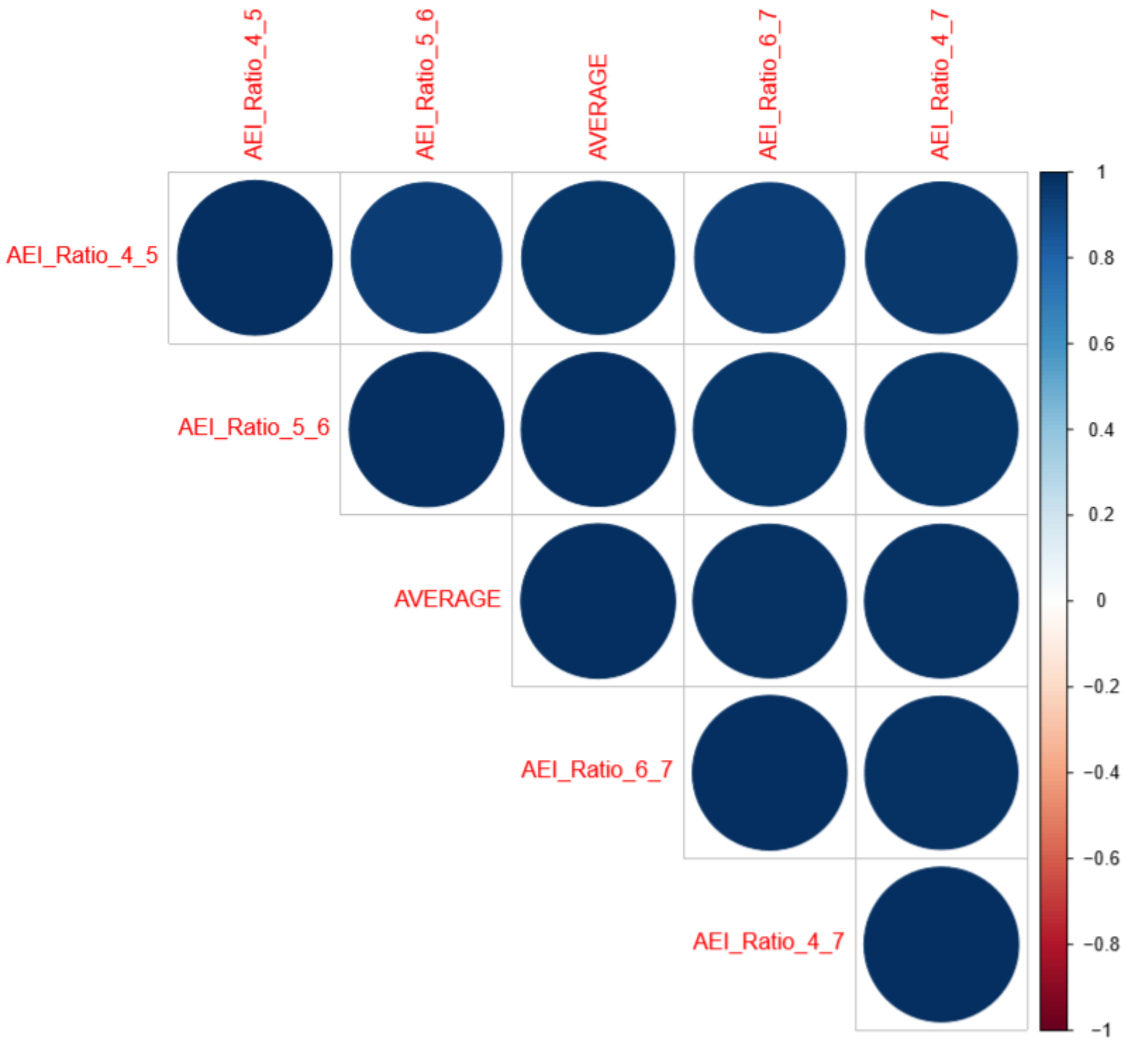

Correlation coefficients

	AEI_Ratio_4_5	AEI_Ratio_5_6	AEI_Ratio_6_7	AEI_Ratio_4_7	AVERAGE
AEI_Ratio_4_5	1.00	0.95	0.94	0.96	0.98
AEI_Ratio_5_6	0.95	1.00	0.98	0.98	0.99
AEI_Ratio_6_7	0.94	0.98	1.00	0.98	0.99
AEI_Ratio_4_7	0.96	0.98	0.98	1.00	0.99
AVERAGE	0.98	0.99	0.99	0.99	1.00

p-values

	AEI_Ratio_4_5	AEI_Ratio_5_6	AEI_Ratio_6_7	AEI_Ratio_4_7	AVERAGE
AEI_Ratio_4_5		0	0	0	0
AEI_Ratio_5_6	0		0	0	0
AEI_Ratio_6_7	0	0		0	0
AEI_Ratio_4_7	0	0	0		0
AVERAGE	0	0	0	0	

BI Low

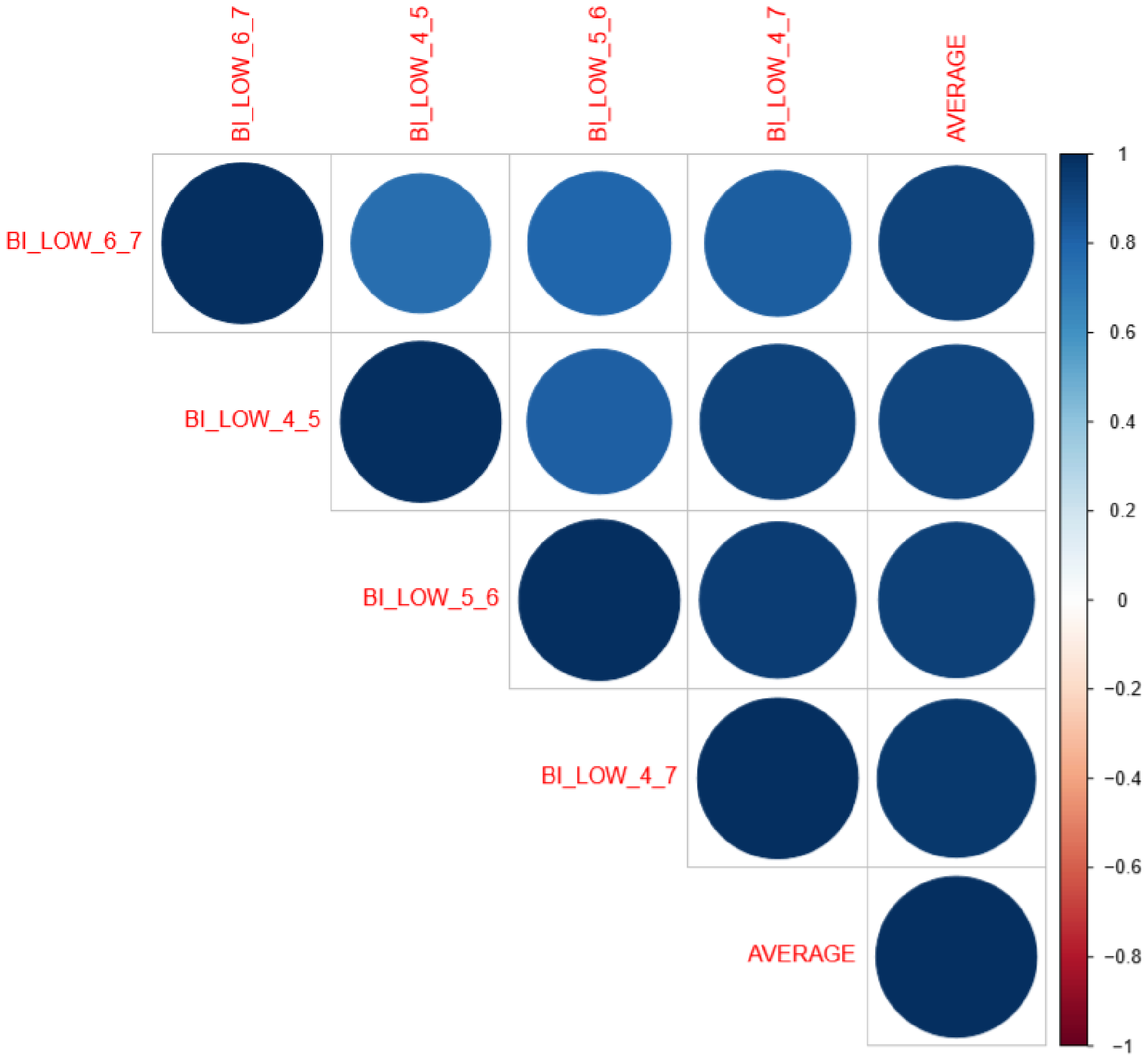

Correlation coefficients

	BI_LOW_4_5	BI_LOW_5_6	BI_LOW_6_7	BI_LOW_4_7	AVERAGE
BI_LOW_4_5	1.00	0.81	0.75	0.93	0.92
BI_LOW_5_6	0.81	1.00	0.79	0.94	0.93
BI_LOW_6_7	0.75	0.79	1.00	0.82	0.92
BI_LOW_4_7	0.93	0.94	0.82	1.00	0.97
AVERAGE	0.92	0.93	0.92	0.97	1.00

p-values

	BI_LOW_4_5	BI_LOW_5_6	BI_LOW_6_7	BI_LOW_4_7	AVERAGE
BI_LOW_4_5		0	0	0	0
BI_LOW_5_6	0		0	0	0
BI_LOW_6_7	0	0		0	0
BI_LOW_4_7	0	0	0		0
AVERAGE	0	0	0	0	

BI High

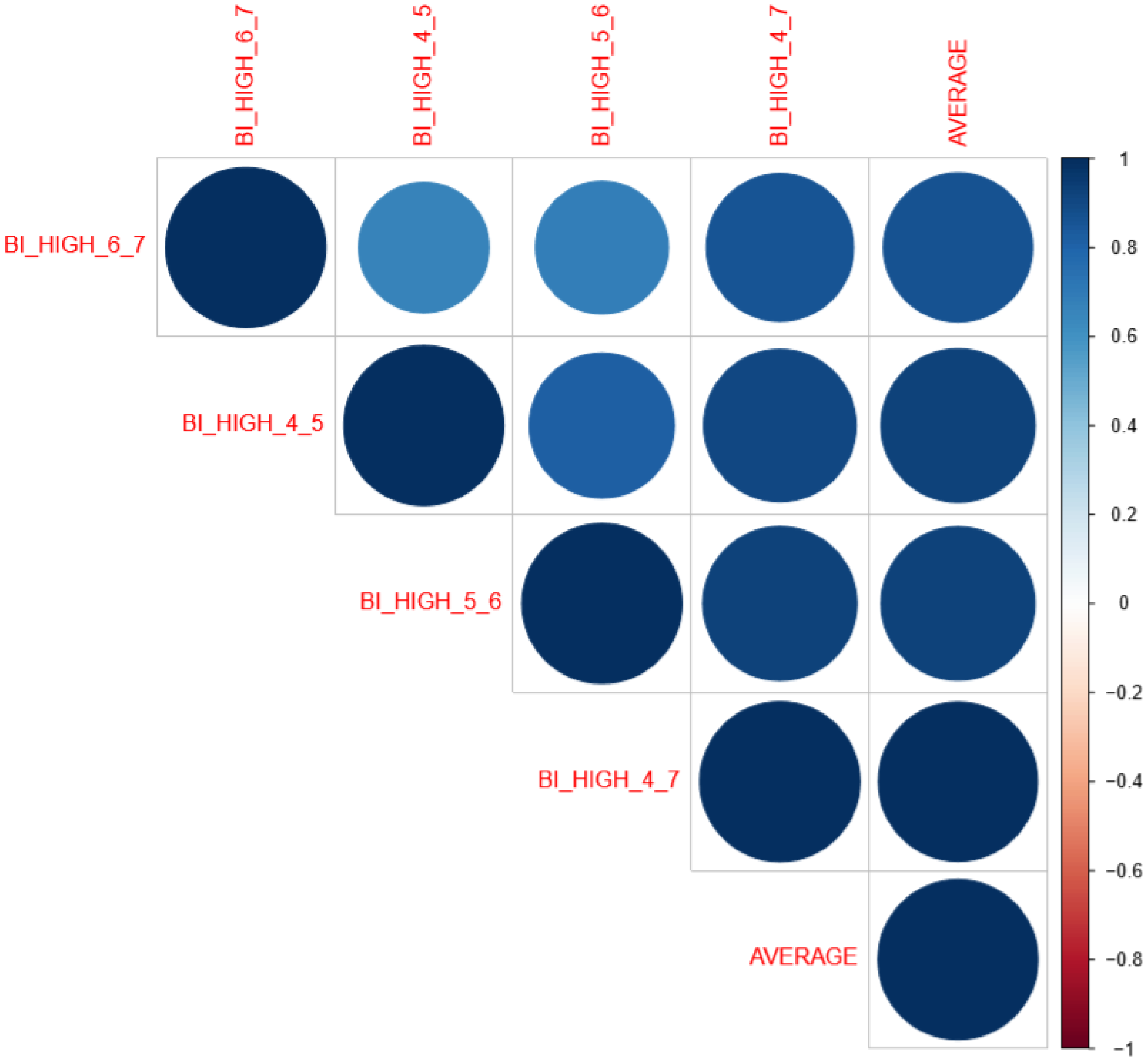

Correlation coefficients

	BI_HIGH_4_5	BI_HIGH_5_6	BI_HIGH_6_7	BI_HIGH_4_7	AVERAGE
BI_HIGH_4_5	1.00	0.82	0.67	0.91	0.92
BI_HIGH_5_6	0.82	1.00	0.69	0.93	0.92
BI_HIGH_6_7	0.67	0.69	1.00	0.85	0.87
BI_HIGH_4_7	0.91	0.93	0.85	1.00	0.99
AVERAGE	0.92	0.92	0.87	0.99	1.00

p-values

	BI_HIGH_4_5	BI_HIGH_5_6	BI_HIGH_6_7	BI_HIGH_4_7	AVERAGE
BI_HIGH_4_5		0	0	0	0
BI_HIGH_5_6	0		0	0	0
BI_HIGH_6_7	0	0		0	0
BI_HIGH_4_7	0	0	0		0
AVERAGE	0	0	0	0	

BI Ratio

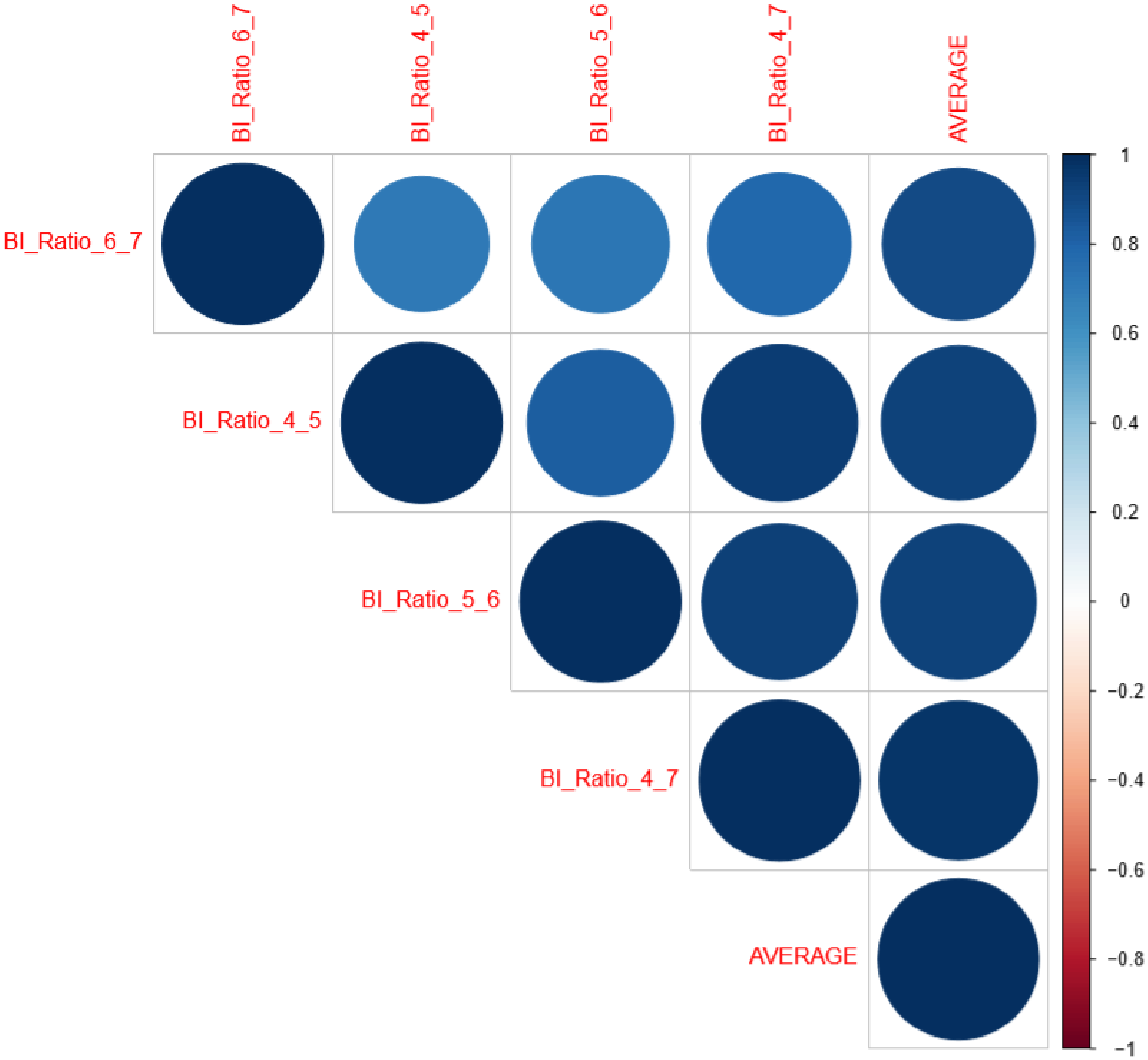

Correlation coefficients

	BI_Ratio_4_5	BI_Ratio_5_6	BI_Ratio_6_7	BI_Ratio_4_7	AVERAGE
BI_Ratio_4_5	1.00	0.83	0.70	0.95	0.92
BI_Ratio_5_6	0.83	1.00	0.73	0.94	0.93
BI_Ratio_6_7	0.70	0.73	1.00	0.79	0.89
BI_Ratio_4_7	0.95	0.94	0.79	1.00	0.97
AVERAGE	0.92	0.93	0.89	0.97	1.00

p-values

	BI_Ratio_4_5	BI_Ratio_5_6	BI_Ratio_6_7	BI_Ratio_4_7	AVERAGE
BI_Ratio_4_5	NA	0	0	0	0
BI_Ratio_5_6	0	NA	0	0	0
BI_Ratio_6_7	0	0	NA	0	0
BI_Ratio_4_7	0	0	0	NA	0
AVERAGE	0	0	0	0	NA

High RMS

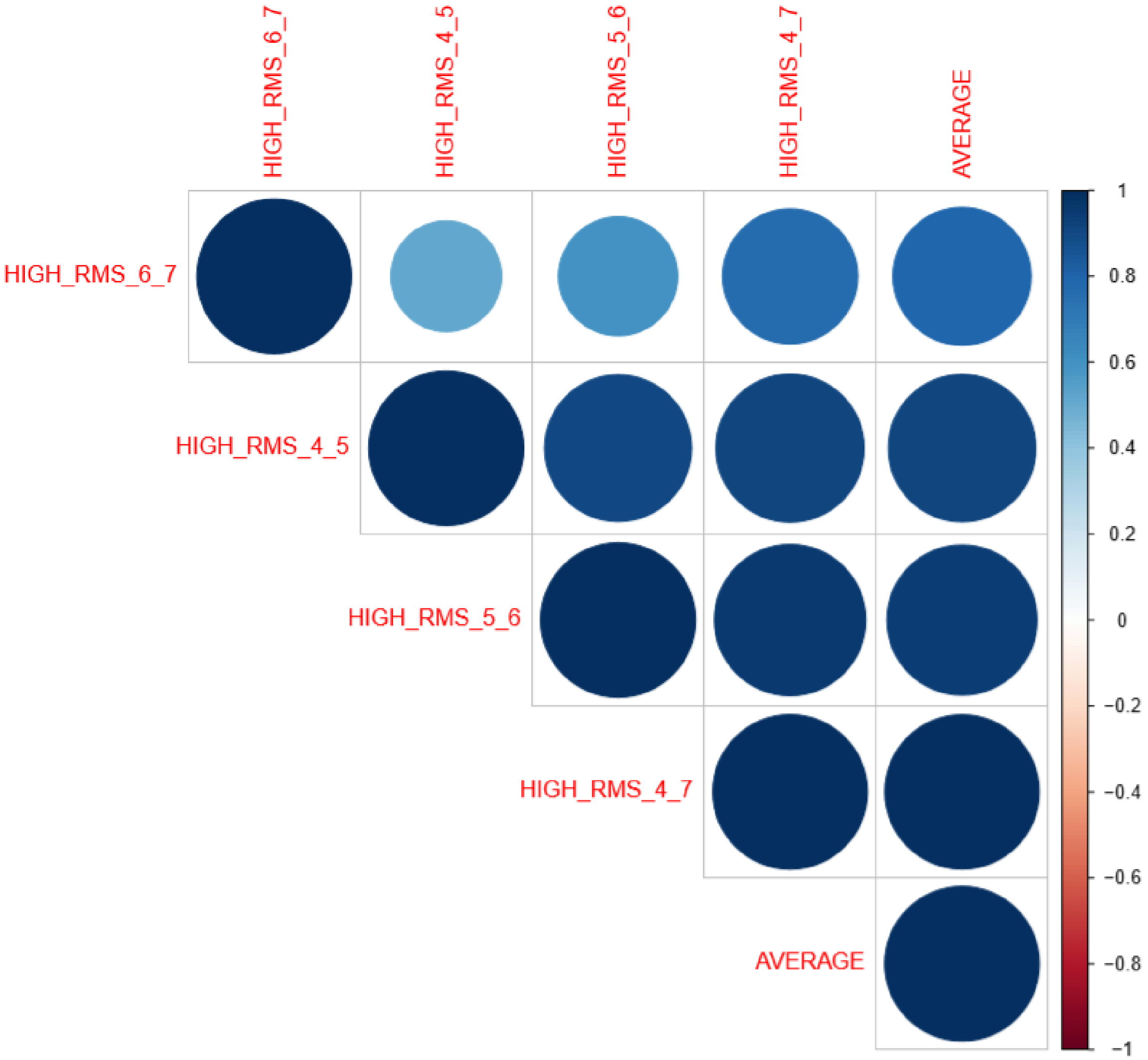

Correlation coefficients

	HIGH_RMS_4_5	HIGH_RMS_5_6	HIGH_RMS_6_7	HIGH_RMS_4_7	AVERAGE
HIGH_RMS_4_5	1.00	0.90	0.52	0.92	0.91
HIGH_RMS_5_6	0.90	1.00	0.59	0.96	0.94
HIGH_RMS_6_7	0.52	0.59	1.00	0.76	0.80
HIGH_RMS_4_7	0.92	0.96	0.76	1.00	1.00
AVERAGE	0.91	0.94	0.80	1.00	1.00

p-values

	HIGH_RMS_4_5	HIGH_RMS_5_6	HIGH_RMS_6_7	HIGH_RMS_4_7	AVERAGE
HIGH_RMS_4_5		0.0000	0.0018	0.0000	0.0000
HIGH_RMS_5_6	0.0000		0.0002	0.0000	0.0000
HIGH_RMS_6_7	0.0018	0.0002		0.0000	0.0000
HIGH_RMS_4_7	0.0000	0.0000	0.0000		0.0000
AVERAGE	0.0000	0.0000	0.0000	0.0000	

Low RMS

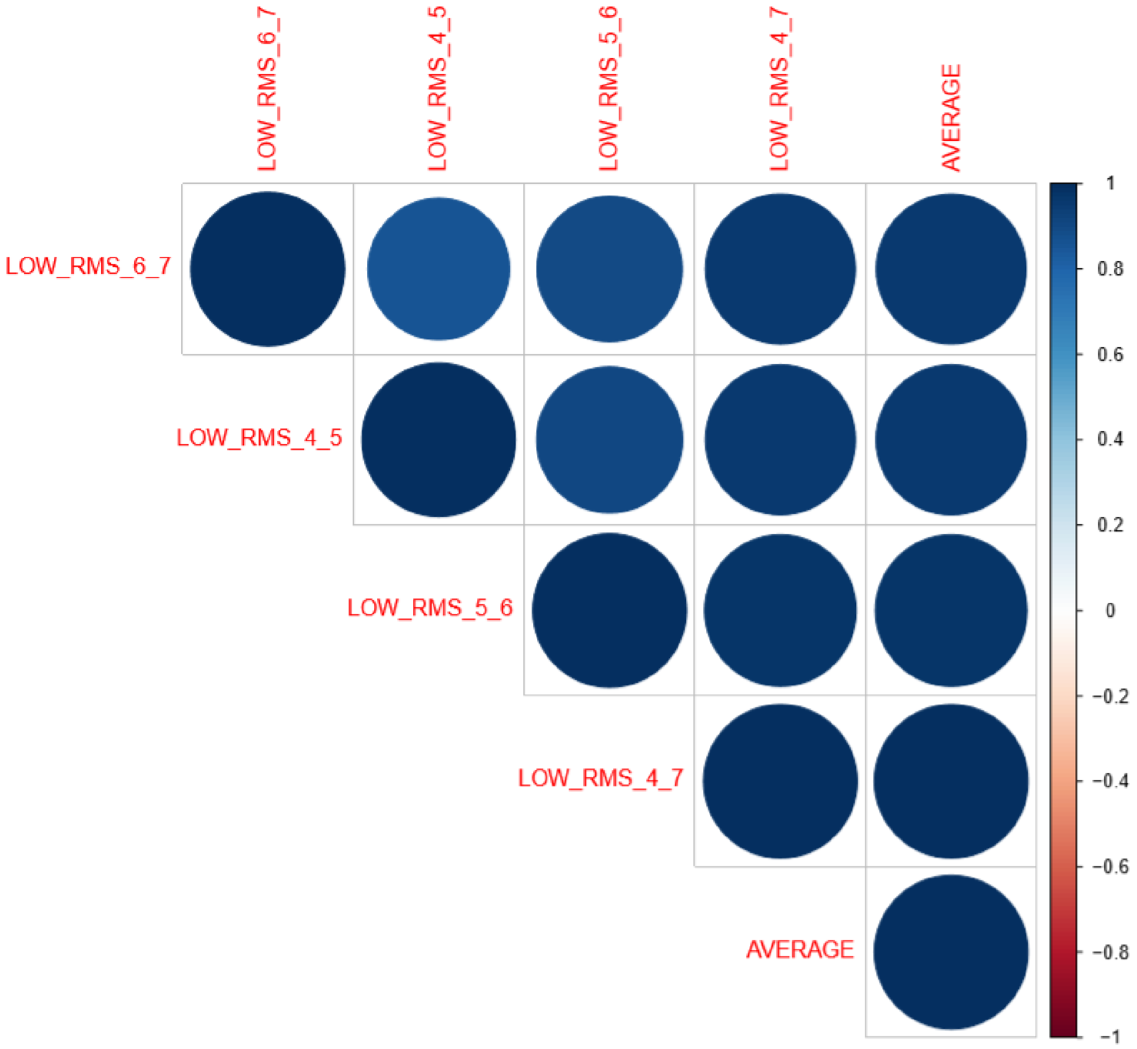

Correlation coefficients

	LOW_RMS_4_5	LOW_RMS_5_6	LOW_RMS_6_7	LOW_RMS_4_7	AVERAGE
LOW_RMS_4_5	1.00	0.91	0.85	0.96	0.96
LOW_RMS_5_6	0.91	1.00	0.90	0.97	0.97
LOW_RMS_6_7	0.85	0.90	1.00	0.95	0.95
LOW_RMS_4_7	0.96	0.97	0.95	1.00	1.00
AVERAGE	0.96	0.97	0.95	1.00	1.00

p-values

	LOW_RMS_4_5	LOW_RMS_5_6	LOW_RMS_6_7	LOW_RMS_4_7	AVERAGE
LOW_RMS_4_5	NA	1.680878e-13	1.925970e-10	0	0
LOW_RMS_5_6	1.680878e-13	NA	7.145395e-13	0	0
LOW_RMS_6_7	1.925970e-10	7.145395e-13	NA	0	0
LOW_RMS_4_7	0.000000e+00	0.000000e+00	0.000000e+00	NA	0
AVERAGE	0.000000e+00	0.000000e+00	0.000000e+00	0	NA

RMS Ratio

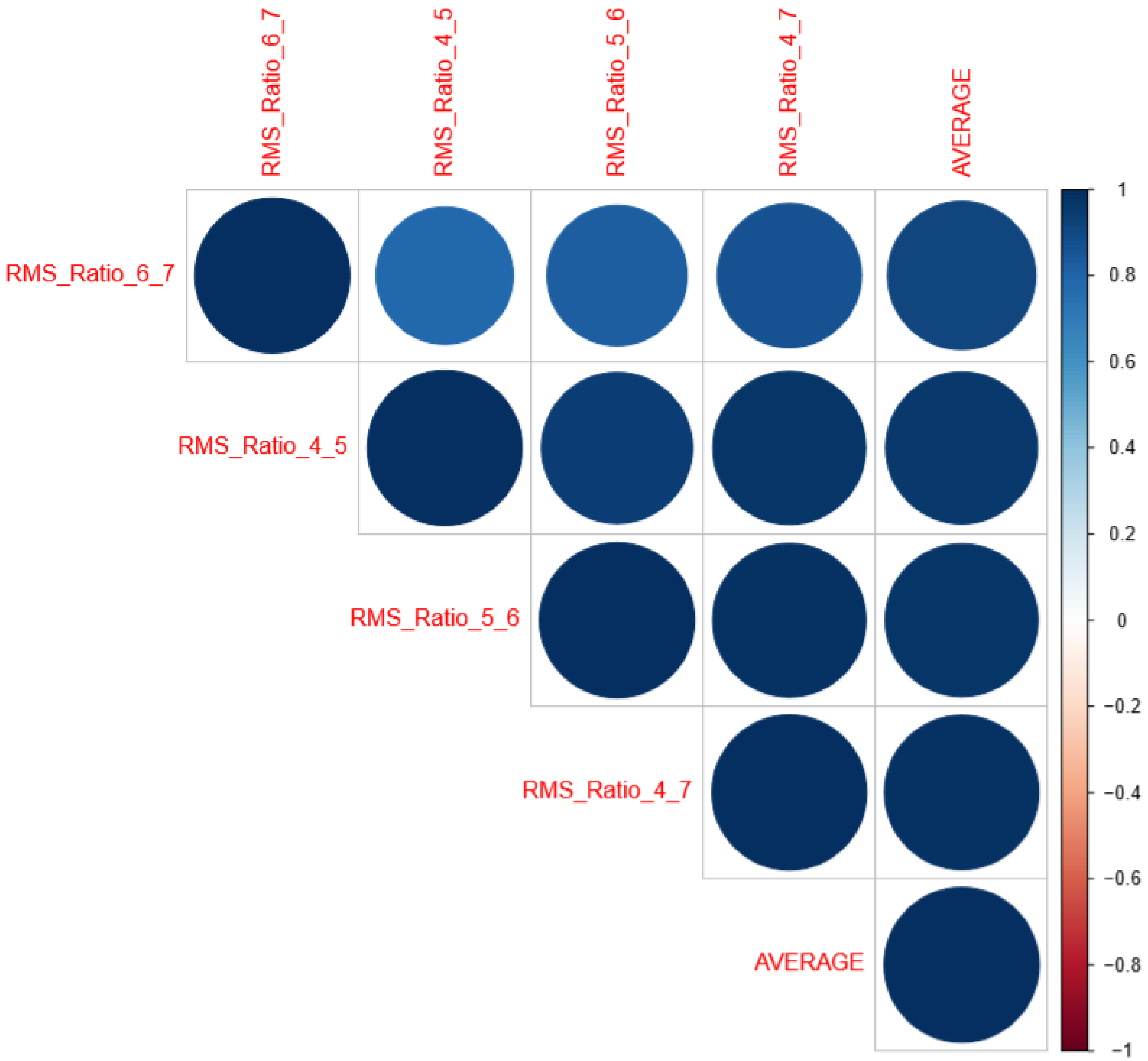

Correlation coefficients

	RMS_Ratio_4_5	RMS_Ratio_5_6	RMS_Ratio_6_7	RMS_Ratio_4_7	AVERAGE
RMS_Ratio_4_5	1.00	0.95	0.79	0.97	0.96
RMS_Ratio_5_6	0.95	1.00	0.82	0.98	0.97
RMS_Ratio_6_7	0.79	0.82	1.00	0.86	0.92
RMS_Ratio_4_7	0.97	0.98	0.86	1.00	0.99
AVERAGE	0.96	0.97	0.92	0.99	1.00

p-values

	RMS_Ratio_4_5	RMS_Ratio_5_6	RMS_Ratio_6_7	RMS_Ratio_4_7	AVERAGE
RMS_Ratio_4_5		0	0	0	0
RMS_Ratio_5_6	0		0	0	0
RMS_Ratio_6_7	0	0		0	0
RMS_Ratio_4_7	0	0	0		0
AVERAGE	0	0	0	0	

High Roughness

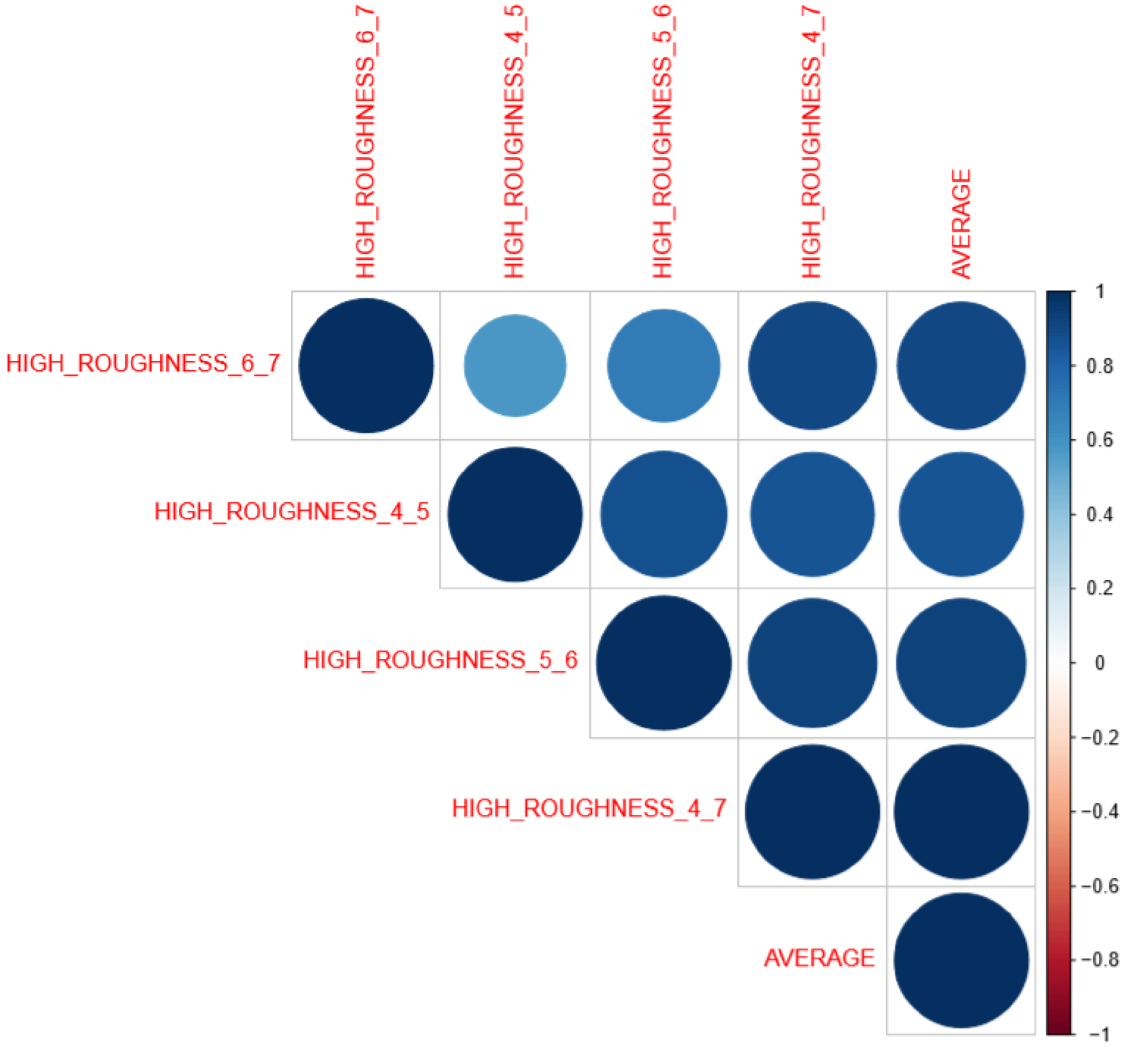

Correlation coefficients

	HIGH_ROUGHNESS_4_5	HIGH_ROUGHNESS_5_6	HIGH_ROUGHNESS_6_7	HIGH_ROUGHNESS_4_7	AVERAGE
HIGH_ROUGHNESS_4_5	1.00	0.88	0.57	0.85	0.85
HIGH_ROUGHNESS_5_6	0.88	1.00	0.70	0.92	0.92
HIGH_ROUGHNESS_6_7	0.57	0.70	1.00	0.90	0.90
HIGH_ROUGHNESS_4_7	0.85	0.92	0.90	1.00	1.00
AVERAGE	0.85	0.92	0.90	1.00	1.00

p-values

	HIGH_ROUGHNESS_4_5	HIGH_ROUGHNESS_5_6	HIGH_ROUGHNESS_6_7	HIGH_ROUGHNESS_4_7	AVERAGE
HIGH_ROUGHNESS_4_5		0e+00	4e-04	0e+00	0e+00
HIGH_ROUGHNESS_5_6	0e+00		0e+00	0e+00	0e+00
HIGH_ROUGHNESS_6_7	4e-04	0e+00		0e+00	0e+00
HIGH_ROUGHNESS_4_7	0e+00	0e+00	0e+00		0e+00
AVERAGE	0e+00	0e+00	0e+00	0e+00	

Low Roughness

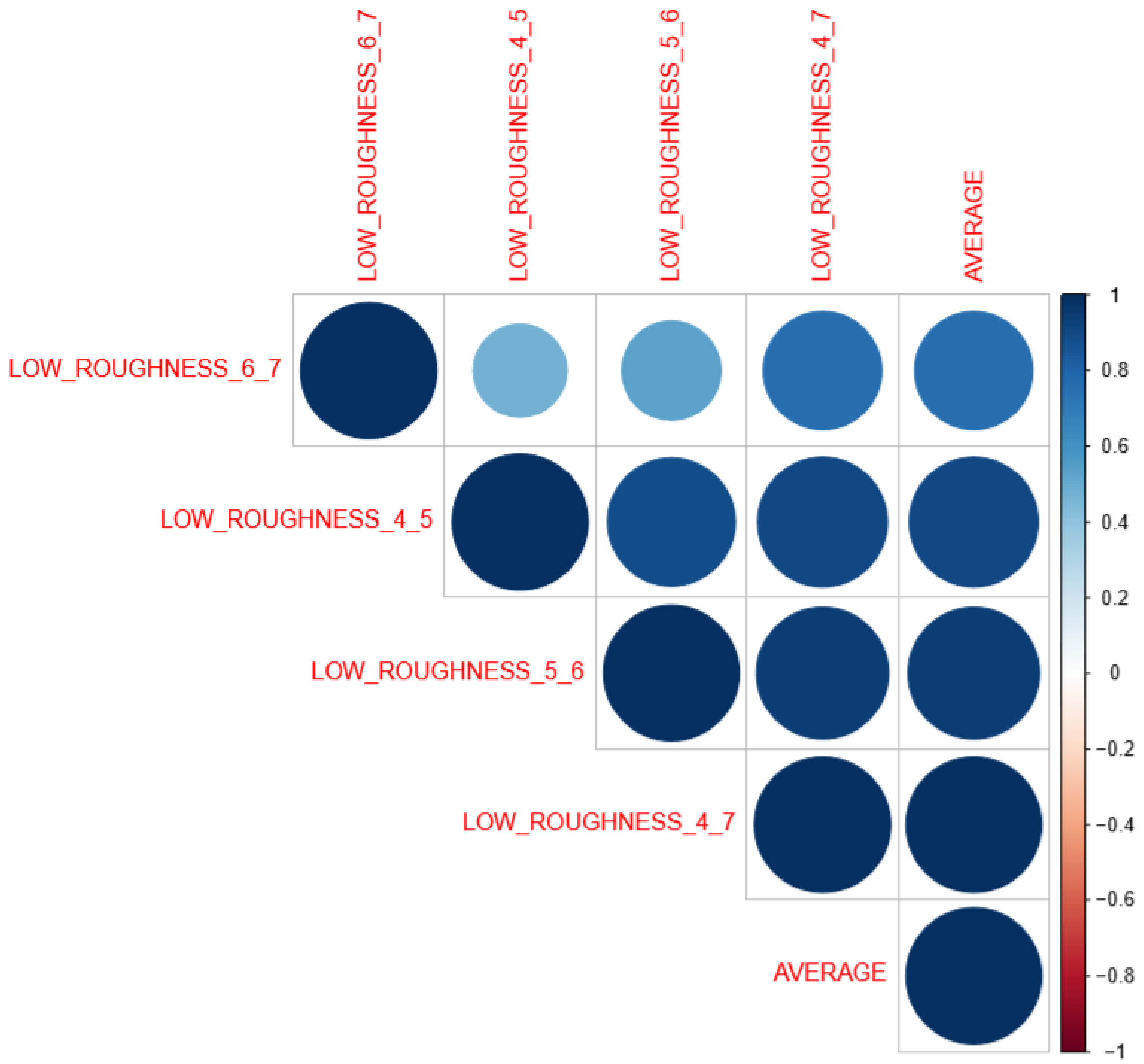

Correlation coefficients

	LOW_ROUGHNESS_4_5	LOW_ROUGHNESS_5_6	LOW_ROUGHNESS_6_7	LOW_ROUGHNESS_4_7	AVERAGE
LOW_ROUGHNESS_4_5	1.00	0.89	0.47	0.91	0.91
LOW_ROUGHNESS_5_6	0.89	1.00	0.54	0.94	0.94
LOW_ROUGHNESS_6_7	0.47	0.54	1.00	0.76	0.76
LOW_ROUGHNESS_4_7	0.91	0.94	0.76	1.00	1.00
AVERAGE	0.91	0.94	0.76	1.00	1.00

p-values

	LOW_ROUGHNESS_4_5	LOW_ROUGHNESS_5_6	LOW_ROUGHNESS_6_7	LOW_ROUGHNESS_4_7	AVERAGE
LOW_ROUGHNESS_4_5		0.0000	0.0047	0.0000	0.0000
LOW_ROUGHNESS_5_6	0.0000		0.0010	0.0000	0.0000
LOW_ROUGHNESS_6_7	0.0047	0.0010		0.0000	0.0000
LOW_ROUGHNESS_4_7	0.0000	0.0000	0.0000		0.0000
AVERAGE	0.0000	0.0000	0.0000	0.0000	

Roughness Ratio

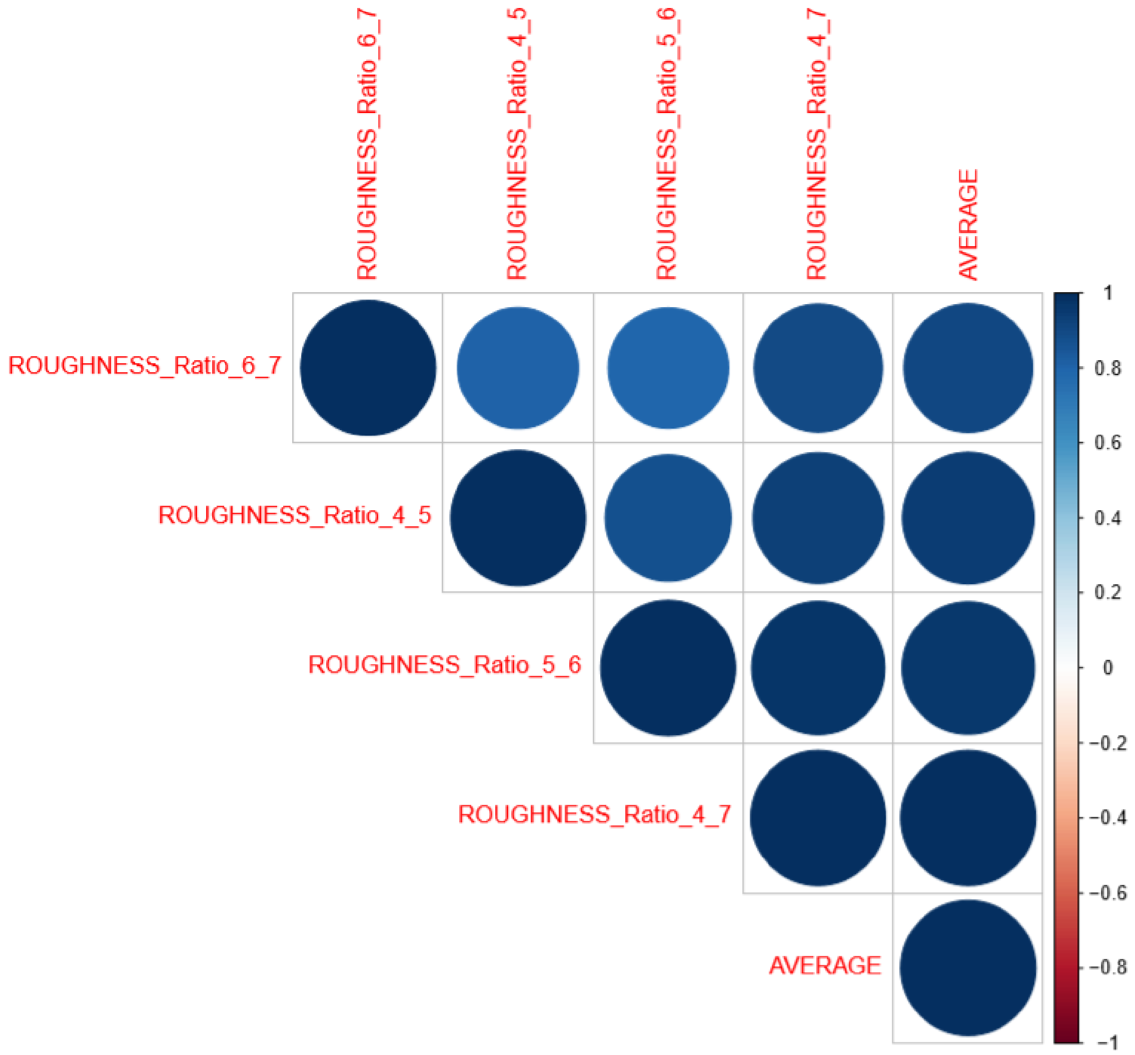

Correlation coefficients

	ROUGHNESS_Ratio_4_5	ROUGHNESS_Ratio_5_6	ROUGHNESS_Ratio_6_7	ROUGHNESS_Ratio_4_7	AVERAGE
ROUGHNESS_Ratio_4_5	1.00	0.87	0.80	0.94	0.95
ROUGHNESS_Ratio_5_6	0.87	1.00	0.80	0.97	0.96
ROUGHNESS_Ratio_6_7	0.80	0.80	1.00	0.90	0.91
ROUGHNESS_Ratio_4_7	0.94	0.97	0.90	1.00	1.00
AVERAGE	0.95	0.96	0.91	1.00	1.00

p-values

	ROUGHNESS_Ratio_4_5	ROUGHNESS_Ratio_5_6	ROUGHNESS_Ratio_6_7	ROUGHNESS_Ratio_4_7	AVERAGE
ROUGHNESS_Ratio_4_5		0	0	0	0
ROUGHNESS_Ratio_5_6	0		0	0	0
ROUGHNESS_Ratio_6_7	0	0		0	0
ROUGHNESS_Ratio_4_7	0	0	0		0
AVERAGE	0	0	0	0	

ACI HIGH

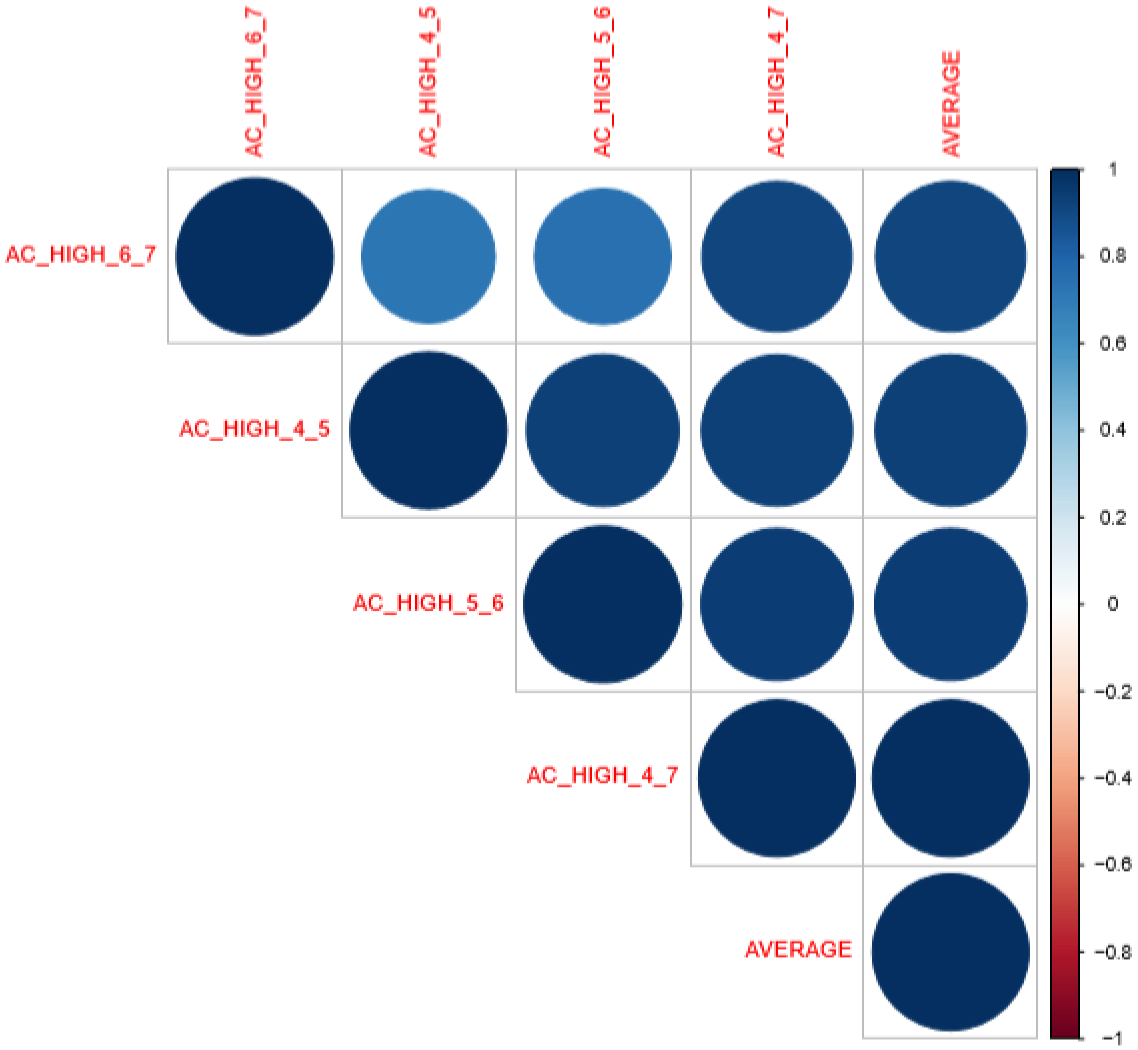

Correlation coefficients

	AC_HIGH_4_5	AC_HIGH_5_6	AC_HIGH_6_7	AC_HIGH_4_7
AC_HIGH_4_5	1.00	0.94	0.73	0.93
AC_HIGH_5_6	0.94	1.00	0.75	0.94
AC_HIGH_6_7	0.73	0.75	1.00	0.92
AC_HIGH_4_7	0.93	0.94	0.92	1.00

p-values

	AC_HIGH_4_5	AC_HIGH_5_6	AC_HIGH_6_7	AC_HIGH_4_7
AC_HIGH_4_5		0	0	0
AC_HIGH_5_6	0		0	0
AC_HIGH_6_7	0	0		0
AC_HIGH_4_7	0	0	0	

AC LOW

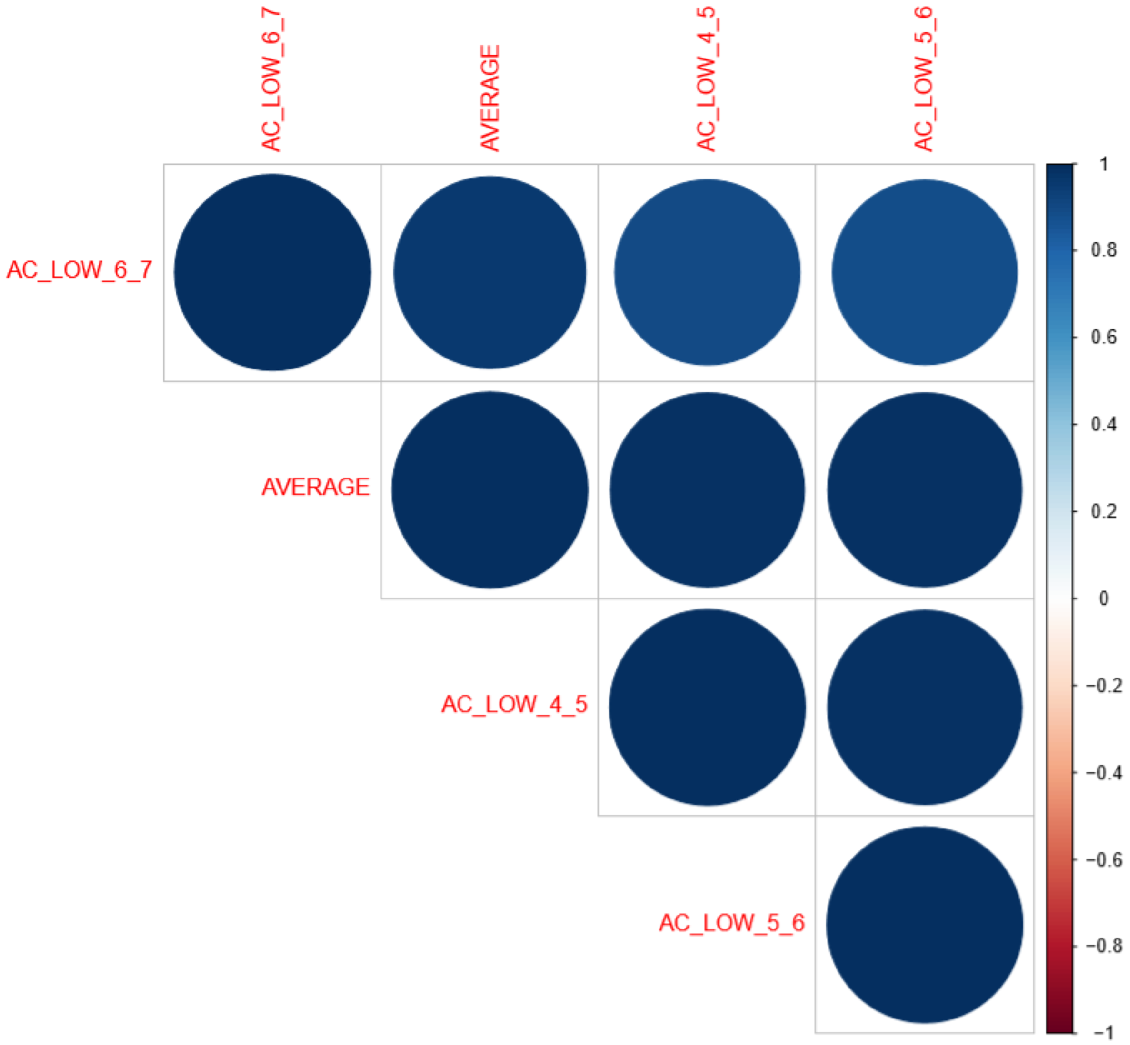

Correlation coefficients

	AC_LOW_4_5	AC_LOW_5_6	AC_LOW_6_7	AVERAGE
AC_LOW_4_5	1.00	0.98	0.89	0.98
AC_LOW_5_6	0.98	1.00	0.89	0.98
AC_LOW_6_7	0.89	0.89	1.00	0.96
AVERAGE	0.98	0.98	0.96	1.00

p-values

	AC_LOW_4_5	AC_LOW_5_6	AC_LOW_6_7	AVERAGE
AC_LOW_4_5		0	0	0
AC_LOW_5_6	0		0	0
AC_LOW_6_7	0	0		0
AVERAGE	0	0	0	

AC Ratio

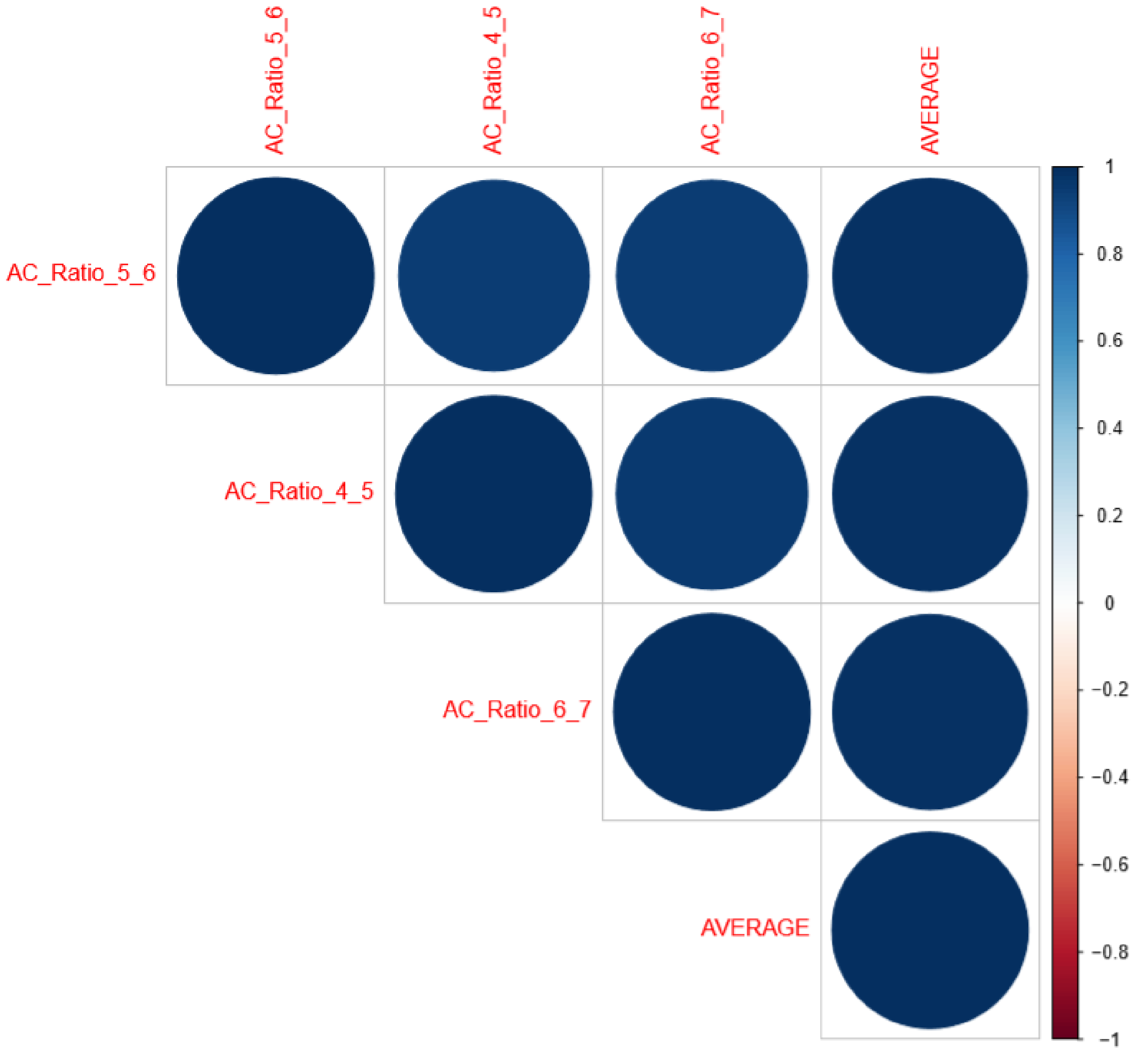

Correlation coefficients

	AC_Ratio_4_5	AC_Ratio_5_6	AC_Ratio_6_7	AVERAGE
AC_Ratio_4_5	1.00	0.94	0.95	0.98
AC_Ratio_5_6	0.94	1.00	0.95	0.98
AC_Ratio_6_7	0.95	0.95	1.00	0.98
AVERAGE	0.98	0.98	0.98	1.00

p-values

	AC_Ratio_4_5	AC_Ratio_5_6	AC_Ratio_6_7	AVERAGE
AC_Ratio_4_5		0	0	0
AC_Ratio_5_6	0		0	0
AC_Ratio_6_7	0	0		0
AVERAGE	0	0	0	
