# Supplementary material for: Rapid coral reef assessment using 3D modelling and acoustics: acoustic indices correlate to fish abundance, diversity and environmental indicators in West Papua, Indonesia
Source: PeerJ. 2021 Feb 8;9:e10761. doi: 10.7717/peerj.10761 (PMC7877240; doi:10.7717/peerj.10761)
Supplement: Supplemental Information 2 [file peerj-09-10761-s002.pdf]

S2. Acoustic Index GLM Model summaries

a) Fish Abundance

Model	Model name	Intercept	Predictor	slope p	Anova null	Deviance explained
14	model_fish_AEI_HIGH	2.357471977	4.342293988	1.1698E-236	7.4002E-282	40.0%
7	model_fish_AC_LOW	8.413984519	-0.030357302	7.766E-235	9.3549E-271	38.4%
3	model_fish_RMS_Ratio	3.443383707	0.063992354	7.8297E-223	5.7563E-236	33.5%
8	model_fish_AC_HIGH	12.3634033	-0.006267225	5.431E-195	1.4121E-232	33.0%
9	model_fish_AC_Ratio	10.11268859	-54.43205162	2.0552E-194	1.3176E-223	31.7%
11	model_fish_AD_HIGH	7.893873292	-1.747025788	5.04E-217	1.3131E-219	31.1%
17	model_fish_BI_HIGH	8.936499752	-0.288649423	1.24E-168	1.0531E-166	23.6%
15	model_fish_AEI_Ratio	5.624791826	-2.789763802	2.7166E-134	1.0848E-164	23.3%
12	model_fish_AD_Ratio	2.377478482	1.708969567	1.2214E-176	1.813E-161	22.8%
1	model_fish_HIGH_RMS	6.397856067	-0.002844679	1.2099E-135	2.2252E-150	21.2%
18	model_fish_BI_Ratio	3.450210798	11.10670437	1.7463E-141	2.0833E-132	18.7%
2	model_fish_LOW_RMS	3.730937767	9.22415E-05	1.1566E-129	4.9422E-126	17.7%
16	model_fish_BI_LOW	3.718879708	0.618948416	1.01597E-67	1.14669E-61	8.5%
5	model_fish_HIGH_ROUGHNESS	5.388788228	-1.05097E-11	3.10681E-47	7.88659E-50	6.9%
6	model_fish_ROUGHNESS_Ratio	4.551031392	0.829609795	3.07976E-17	2.04343E-16	2.1%
10	model_fish_AD_LOW	-3.908771555	3.56520779	7.97072E-13	5.29476E-13	1.6%
4	model_fish_LOW_ROUGHNESS	4.584453052	1.20625E-11	1.76453E-11	5.59234E-11	1.3%
13	model_fish_AEI_LOW	4.848573324	-0.794371976	0.015186739	0.015407269	0.2%

b) Fish Species Diversity

Model	Model name	Intercept	Predictor	slope p	Anova null	Deviance explained
1	model_fish_HIGH_RMS	6.397856	-0.002844679	1.2099E-135	2.2252E-150	0.212212698
2	model_fish_LOW_RMS	3.730938	9.22415E-05	1.1566E-129	4.9422E-126	0.177390762
3	model_fish_RMS_Ratio	3.443384	0.063992354	7.8297E-223	5.7563E-236	0.33467873
4	model_fish_LOW_ROUGHNESS	4.584453	1.20625E-11	1.76453E-11	5.59234E-11	0.013363116
5	model_fish_HIGH_ROUGHNESS	5.388788	-1.05097E-11	3.10681E-47	7.88659E-50	0.068520883
6	model_fish_ROUGHNESS_Ratio	4.551031	0.829609795	3.07976E-17	2.04343E-16	0.021016172
7	model_fish_AC_LOW	8.413985	-0.030357302	7.766E-235	9.3549E-271	0.384472568
8	model_fish_AC_HIGH	12.3634	-0.006267225	5.431E-195	1.4121E-232	0.329827343
9	model_fish_AC_Ratio	10.11269	-54.43205162	2.0552E-194	1.3176E-223	0.316989879
10	model_fish_AD_LOW	-3.90877	3.56520779	7.97072E-13	5.29476E-13	0.016204555
11	model_fish_AD_HIGH	7.893873	-1.747025788	5.04E-217	1.3131E-219	0.311267493
12	model_fish_AD_Ratio	2.377478	1.708969567	1.2214E-176	1.813E-161	0.228075768
13	model_fish_AEI_LOW	4.848573	-0.794371976	0.015186739	0.015407269	0.001825777
14	model_fish_AEI_HIGH	2.357472	4.342293988	1.1698E-236	7.4002E-282	0.400363797
15	model_fish_AEI_Ratio	5.624792	-2.789763802	2.7166E-134	1.0848E-164	0.23268669
16	model_fish_BI_LOW	3.71888	0.618948416	1.01597E-67	1.14669E-61	0.085410543
17	model_fish_BI_HIGH	8.9365	-0.288649423	1.24E-168	1.0531E-166	0.235566454
18	model_fish_BI_Ratio	3.450211	11.10670437	1.7463E-141	2.0833E-132	0.186507927

c) Fish > 30cm length

Model	Model name	Intercept	Predictor	slope p	Anova null	Deviance explained
1	model_fish_HIGH_RMS	2.538073	-0.001338674	0.004836537	0.003899941	2.1%
2	model_fish_LOW_RMS	0.070592	0.000145222	2.64423E-17	2.94563E-17	18.3%
3	model_fish_RMS_Ratio	0.46802	0.062748558	2.96956E-12	7.43662E-13	13.2%
4	model_fish_LOW_ROUGHNESS	1.422775	2.43109E-11	0.001154191	0.001943034	2.5%
5	model_fish_HIGH_ROUGHNESS	1.940599	-3.34171E-12	0.280495189	0.27716012	0.3%
6	model_fish_ROUGHNESS_Ratio	1.577994	0.691006872	0.122264283	0.130843987	0.6%
7	model_fish_AC_LOW	5.641112	-0.032373718	2.41031E-14	1.29182E-16	17.5%
8	model_fish_AC_HIGH	13.04746	-0.009391023	3.18913E-17	2.52295E-23	25.3%
9	model_fish_AC_Ratio	6.087142	-43.83561536	2.67385E-08	4.29675E-09	8.8%
10	model_fish_AD_LOW	-29.4125	12.82466821	1.10525E-07	3.42456E-08	7.8%
11	model_fish_AD_HIGH	4.059138	-1.270842002	2.63057E-07	2.98158E-07	6.7%
12	model_fish_AD_Ratio	0.220252	1.112039559	8.7918E-05	0.000155515	3.7%
13	model_fish_AEI_LOW	2.623153	-6.128954842	2.19437E-05	2.29536E-05	4.6%
14	model_fish_AEI_HIGH	-0.15345	3.501070702	4.77153E-10	3.13853E-11	11.3%
15	model_fish_AEI_Ratio	2.987779	-4.188454058	9.29878E-13	7.14882E-17	17.8%
16	model_fish_BI_LOW	-0.02879	1.042310346	5.06901E-13	2.58433E-11	11.4%
17	model_fish_BI_HIGH	6.727298	-0.345096682	2.36153E-13	2.22274E-13	13.8%
18	model_fish_BI_Ratio	-0.15482	15.81407937	1.81183E-16	1.67704E-15	16.2%

d) Coral Health indicators

Model	Model name	Intercept	Predictor	slope p	Anova null	Deviance explained
1	model_fish_HIGH_RMS	0.308023	-0.001073722	0.416134013	0.407227262	0.014574326
2	model_fish_LOW_RMS	-0.55045	1.98924E-05	0.693004049	0.695004103	0.00326168
3	model_fish_RMS_Ratio	-0.83381	0.026261427	0.280207797	0.281925863	0.024565973
4	model_fish_LOW_ROUGHNESS	-0.28607	-6.02214E-12	0.814001897	0.811467392	0.001207273
5	model_fish_HIGH_ROUGHNESS	0.543384	-1.4459E-11	0.129952985	0.114255568	0.052925411
6	model_fish_ROUGHNESS_Ratio	-0.39314	0.208478861	0.874761686	0.875633501	0.000519725
7	model_fish_AC_LOW	-0.31852	-0.000255358	0.980126834	0.98012794	1.31644E-05
8	model_fish_AC_HIGH	0.466691	-0.000656085	0.765196799	0.764426812	0.001905467
9	model_fish_AC_Ratio	-0.64285	2.838510771	0.880919376	0.881253371	0.000473473
10	model_fish_AD_LOW	5.59185	-2.458382642	0.685957822	0.68724467	0.003439126
11	model_fish_AD_HIGH	1.986886	-1.271174604	0.067757964	0.069019514	0.07015105
12	model_fish_AD_Ratio	-2.10904	1.295194429	0.099772808	0.11375642	0.053071694
13	model_fish_AEI_LOW	-0.70134	2.18823008	0.608061119	0.605075707	0.005673899
14	model_fish_AEI_HIGH	-1.85909	2.857187277	0.061162058	0.049569327	0.081816386
15	model_fish_AEI_Ratio	0.155439	-1.467563523	0.23829149	0.213669257	0.032811477
16	model_fish_BI_LOW	-0.02184	-0.214127673	0.692323315	0.688362723	0.003413222
17	model_fish_BI_HIGH	1.824662	-0.147039986	0.269362841	0.274763171	0.025309219
18	model_fish_BI_Ratio	-0.44447	0.868312525	0.888438658	0.888861157	0.000414364

e) Fishing Pressure indicators

Model	Model name	Intercept	Predictor	slope p	Anova null	Deviance explained
1	model_fish_HIGH_RMS	-1.00631	0.000749434	0.568481879	0.572835579	0.005567322
2	model_fish_LOW_RMS	-2.08636	0.000138431	0.008502727	0.00877061	0.120271551
3	model_fish_RMS_Ratio	-1.40574	0.045874392	0.087197071	0.084756373	0.052024938
4	model_fish_LOW_ROUGHNESS	-0.89047	2.92465E-11	0.190843873	0.215724001	0.026834823
5	model_fish_HIGH_ROUGHNESS	-1.12362	8.58479E-12	0.341931837	0.345872425	0.015557899
6	model_fish_ROUGHNESS_Ratio	-0.62983	0.520124215	0.709670709	0.714406594	0.002344783
7	model_fish_AC_LOW	1.91856	-0.019697502	0.094894491	0.085420268	0.051803986
8	model_fish_AC_HIGH	7.637812	-0.00670674	0.023703672	0.012519242	0.109185567
9	model_fish_AC_Ratio	1.563097	-20.64735835	0.353734232	0.342568998	0.0157724
10	model_fish_AD_LOW	-20.1345	8.088189873	0.254931081	0.245116044	0.023653994
11	model_fish_AD_HIGH	1.791985	-1.258164154	0.097069789	0.098668749	0.04774705
12	model_fish_AD_Ratio	-1.91453	1.032925652	0.239108253	0.255379318	0.022650819
13	model_fish_AEI_LOW	0.098336	-4.136651901	0.35128254	0.353879622	0.01504931
14	model_fish_AEI_HIGH	-2.27414	3.285402325	0.053970234	0.041032232	0.073096401
15	model_fish_AEI_Ratio	0.369568	-2.765067997	0.075602075	0.049003231	0.067853358
16	model_fish_BI_LOW	-1.19785	0.42081739	0.413977247	0.428805433	0.010961908
17	model_fish_BI_HIGH	2.690121	-0.218684891	0.128448322	0.132535547	0.039616027
18	model_fish_BI_Ratio	-1.28365	6.822141846	0.277726541	0.289900885	0.019612011

f) Algal Control indicators

Model	Model name	Intercept	Predictor	slope p	Anova null	Deviance explained
1	model_fish_HIGH_RMS	2.167579	-0.001263214	0.02331643	0.020339986	0.037649768
2	model_fish_LOW_RMS	0.149494	0.000113176	1.72766E-08	2.48238E-08	0.217366387
3	model_fish_RMS_Ratio	0.311787	0.055164251	1.20103E-07	7.19896E-08	0.202924439
4	model_fish_LOW_ROUGHNESS	1.059823	2.70025E-11	0.001847164	0.00311092	0.061143948
5	model_fish_HIGH_ROUGHNESS	2.275573	-1.42027E-11	0.000355279	0.000198396	0.096852566
6	model_fish_ROUGHNESS_Ratio	1.021867	1.667401891	0.000518753	0.000962618	0.076231041
7	model_fish_AC_LOW	3.827045	-0.019611377	1.40983E-05	7.78572E-06	0.139826077
8	model_fish_AC_HIGH	5.431397	-0.003273105	0.000848416	0.000570886	0.083019339
9	model_fish_AC_Ratio	5.379683	-39.91039586	1.26146E-05	4.5533E-06	0.147008311
10	model_fish_AD_LOW	-14.7665	6.668168288	0.013212296	0.011783433	0.044369679
11	model_fish_AD_HIGH	4.047252	-1.449136926	6.49891E-07	6.73487E-07	0.172700337
12	model_fish_AD_Ratio	-0.60499	1.466805228	6.02732E-06	1.44963E-05	0.1315225
13	model_fish_AEI_LOW	2.00124	-4.026503723	0.017908308	0.018539798	0.038782478
14	model_fish_AEI_HIGH	-0.45478	3.458419094	1.61133E-07	2.39328E-08	0.217862709
15	model_fish_AEI_Ratio	2.39742	-3.163074403	3.60366E-07	1.04163E-08	0.229165963
16	model_fish_BI_LOW	-0.06991	0.883714124	5.38317E-07	2.89719E-06	0.153072224
17	model_fish_BI_HIGH	4.133793	-0.185739419	0.000777628	0.0008927	0.077208467
18	model_fish_BI_Ratio	0.057177	11.61806477	4.87845E-07	1.16119E-06	0.165362301
